# Transcriptional Regulation and Mechanism of SigN (ZpdN), a pBS32-Encoded Sigma Factor in Bacillus subtilis

**DOI:** 10.1128/mBio.01899-19

**Published:** 2019-09-17

**Authors:** Aisha T. Burton, Aaron DeLoughery, Gene-Wei Li, Daniel B. Kearns

**Affiliations:** a Department of Biology, Indiana University, Bloomington, Indiana, USA; b Department of Biology, Massachusetts Institute of Technology, Cambridge, Massachusetts, USA; Yale School of Medicine

**Keywords:** sigma factor, RNAP, plasmid, LexA, cell death, RNA polymerases, plasmids, sigma factors

## Abstract

Laboratory strains of Bacillus subtilis encode many alternative sigma factors, each dedicated to expressing a unique regulon such as those involved in stress resistance, sporulation, and motility. The ancestral strain of B. subtilis also encodes an additional sigma factor homolog, ZpdN, not found in lab strains due to being encoded on the large, low-copy-number plasmid pBS32, which was lost during domestication. DNA damage triggers pBS32 hyperreplication and cell death in a manner that depends on ZpdN, but how ZpdN mediates these effects is unknown. Here, we show that ZpdN is a bona fide sigma factor that can direct RNA polymerase to transcribe ZpdN-dependent genes, and we rename ZpdN SigN accordingly. Rend-seq (end-enriched transcriptome sequencing) analysis was used to determine the SigN regulon on pBS32, and the 5′ ends of transcripts were used to predict the SigN consensus sequence. Finally, we characterize the regulation of SigN itself and show that it is transcribed by at least three promoters: *P_sigN1_*, a strong SigA-dependent LexA-repressed promoter; *P_sigN2_*, a weak SigA-dependent constitutive promoter; and *P_sigN3_*, a SigN-dependent promoter. Thus, in response to DNA damage SigN is derepressed and then experiences positive feedback. How cells die in a pBS32-dependent manner remains unknown, but we predict that death is the product of expressing one or more genes in the SigN regulon.

## Abstract (precis)

Sigma factors are utilized by bacteria to control and regulate gene expression. Some sigma factors are activated during times of stress to ensure the survival of the bacterium. Here, we report the presence of a sigma factor that is encoded on a plasmid that leads to cellular death after DNA damage.

## INTRODUCTION

Propagation and cultivation of bacteria in the laboratory have been shown to select for enhanced axenic growth and genetic tractability in a process called domestication. The model genetic organism Bacillus subtilis is an example of a commonly-used domesticated bacterium, as the laboratory strains differ substantially from the ancestor from which they were derived. For example, lab strains are defective for biofilm formation, are reduced in motility, are auxotrophic for one or more amino acids, and are deficient in the ability to synthesize multiple antibiotics, a potent surfactant, and a viscous slime layer (1–5). While many traits were lost during the domestication of laboratory strains, one important trait was gained: high-frequency uptake of extracellular DNA in a process called natural genetic competence. Later, it was shown that increased genetic competence was also due to genetic loss, in this case due to the loss of the endogenous plasmid pBS32 (6, 7).

pBS32 is a large, 84-kb, low-copy-number plasmid that has a separate replication initiation protein and a high-fidelity plasmid partitioning system (6, 8–10). Moreover, pBS32 has been shown to encode an inhibitor of competence for DNA uptake (ComI) (7) and an inhibitor of biofilm formation (RapP) that regulates cell physiology (11–13). In addition, approximately one-third of the pBS32 sequence encodes a cryptic prophage-like element, and cell death is triggered in a pBS32-dependent manner following treatment with the DNA-damaging agent mitomycin C (MMC) (7, 14–17). pBS32-dependent cell death upon mitomycin C treatment requires a plasmid-encoded sigma factor homolog, ZpdN, and artificial ZpdN induction was shown to be sufficient to trigger cell death (17). How ZpdN is activated by the presence of DNA damage and the mechanism by which ZpdN promotes cell death are unknown.

Here, we show that ZpdN functions as a bona fide sigma factor which directs RNA polymerase to transcribe a large regulon of genes carried on pBS32. Based on our findings, we rename ZpdN SigN and propose a SigN-dependent consensus sequence for transcriptional activation. We show that SigN induction triggers immediate loss of cell viability, even as cells continue to grow and the cell culture increases in optical density (OD). We characterize the *sigN* promoter region and find multiple promoters that activate its expression, including a DNA damage-responsive LexA-repressed promoter and a separate promoter that governs autoactivation. Finally, the SigN regulon does not appear to include the pBS32 putative prophage region, and thus, cell death may be prophage independent. The gene or genes responsible for pBS32-mediated cell death remain unknown, but we infer that they must reside within the plasmid, expressed by RNA polymerase and SigN.

## RESULTS

### SigN is repressed by LexA.

**FIG 1:**
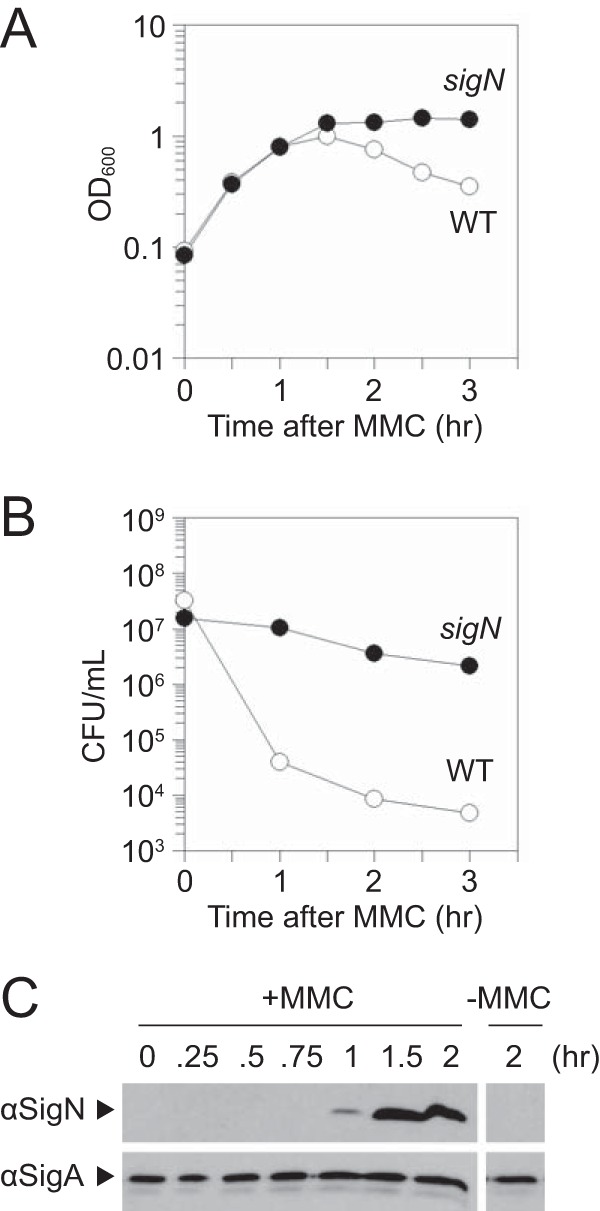
SigN is required for loss of cell viability after MMC treatment. (A) Optical density (OD_600_) growth curve of wild type (open circles, DK607) and *sigN* mutant (closed circles, DK3287). The *x* axis is the time of spectrophotometry after MMC addition. (B) CFU growth curve of wild type (open circles, DK607) and *sigN* mutant (closed circles, DK3287). The *x* axis is the time of dilution plating after MMC addition. (C) Western blot analysis of wild-type DK607 cell lysates harvested at the indicated time after MMC addition and probed with either anti-SigN antibody or anti-SigA antibody. On the right is a single panel of the same strain for comparison 2 h after mock MMC addition.

SigN (formerly ZpdN) is a sigma factor homolog encoded on the plasmid pBS32 that is necessary and sufficient for pBS32-mediated cell death (17). Consistent with previous results, treatment of cells deleted for the PBSX and SPβ prophages (14, 18–21) with the DNA-damaging agent mitomycin C (MMC) caused a 3-fold decrease in optical density (OD) from peak absorbance, and the decrease in OD was abolished in cells deleted for *sigN* (17) (Fig. 1A). To determine the effect of MMC on cell viability, viable counting was performed by dilution plating over a time course following MMC addition. Addition of MMC caused a rapid and immediate decline in CFU such that the number of viable cells decreased 3 orders of magnitude even as the OD increased for three doublings (compare Fig. 1A and B). As with loss of OD, mutation of *sigN* abolished the MMC-dependent decrease in cell viability (Fig. 1B). We conclude that pBS32-mediated cell death occurs prior to, and independently of, transient cell growth and the subsequent decline in OD. We further conclude that SigN is required for all pBS32-dependent death-related phenotypes thus far observed.

**FIG 2:**
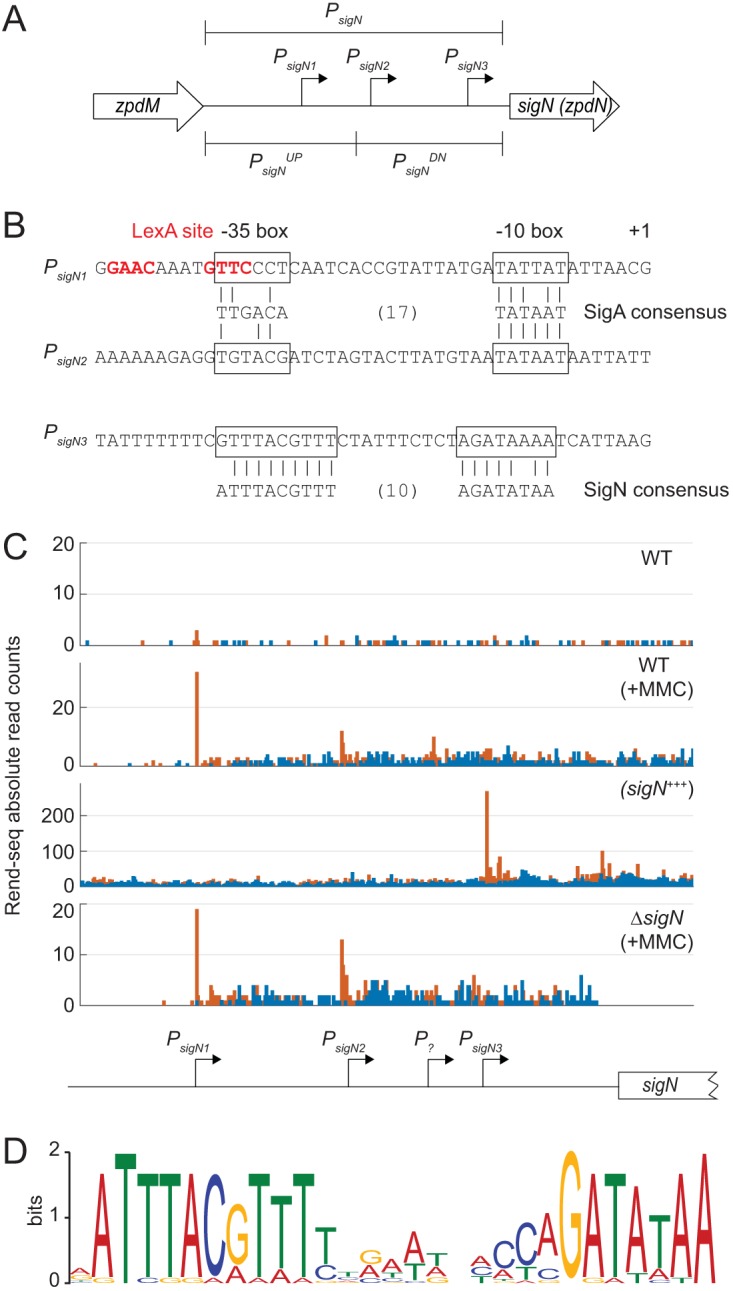
The *sigN* promoter region. (A) A schematic of the promoter region of *sigN*. Open arrows indicate reading frames. Bent arrows indicate promoters. Promoter regions are indicated by brackets. (B) Promoter sequences. Boxes surround −35 and −10 regions relative to the +1 transcriptional start site. Below the promoters are SigA and SigN consensus sequences with vertical lines to indicate a consensus match. (C) Rend-seq data for the indicated genotypes: WT (DK607), WT+MMC (DK607 induced for 2 h with MMC), *sigN*^+++^ (DK1634 induced for 1 h with 1 mM IPTG), and *ΔsigN*+MMC (DK3287 induced for 2 h with MMC). Orange peaks represent absolute read counts for 5′ ends, and blue peaks represent absolute read counts for 3′ ends. Below is a cartoon indicating the location of the promoter believed to be responsible for the transcriptional start sites predicted above relative to the *sigN* coding region. Note that the peaks stop abruptly in the last panel due to deletion of the *sigN* gene. Information on Rend-seq is included in Table S3. Data that mapped to the plasmid in Fig. S2 are represented after normalization per million reads. (D) SigN consensus sequence generated by MEME sequence analysis using the promoters listed in Table 2.

**FIG 3:**
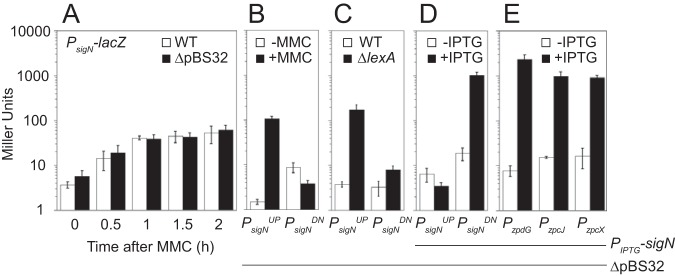
The *sigN* promoter region is repressed by LexA and autoactivated. (A) β-Galactosidase activity of a *P_sigN_-lacZ* reporter in the presence (open bars) and absence (closed bars) of pBS32 measured at the indicated time points following 800 nM MMC addition. The following strains were used to generate this panel: DK4784 (WT) and DK5066 (ΔpBS32). (B) β-Galactosidase activity of either a *P_sigN_^UP^-lacZ* or *P_sigN_^DN^-lacZ* reporter in the presence (closed bars) and absence (open bars) of 800 nM MMC (1 h incubation). The following strains were used to generate this panel: DK5657 (*P_sigN_^UP^-lacZ* ΔpBS32) and DK5658 (*P_sigN_^DN^-lacZ* ΔpBS32). (C) β-Galactosidase activity of either a *P_sigN_^UP^-lacZ* or *P_sigN_^DN^-lacZ* reporter in the presence (open bars) and absence (closed bars) of LexA. The following strains were used to generate this panel: DK7291 (*P_sigN_^UP^-lacZ* ΔpBS32), DK7292 (*P_sigN_^DN^-lacZ* ΔpBS32), DK7259 *P_sigN_^UP^-lacZ* ΔpBS32 *lexA*), and DK7260 (*P_sigN_^DN^-lacZ* ΔpBS32 *lexA*). (D) β-Galactosidase activity of either a *P_sigN_^UP^-lacZ* or *P_sigN_^DN^-lacZ* reporter in a strain containing an IPTG-inducible SigN construct grown in the presence (closed bars) and absence (open bars) of 1 mM IPTG. The following strains were used to generate this panel: DK5657 (*P_sigN_^UP^-lacZ* ΔpBS32) and DK5658 (*P_sigN_^DN^-lacZ* ΔpBS32). (E) β-Galactosidase activity of a *P_zpdG_-lacZ*, *P_zpcJ_-lacZ*, or *P_zpcX_-lacZ* reporter in a strain containing an IPTG-inducible SigN construct grown in the presence (closed bars) and absence (open bars) of 1 mM IPTG. The following strains were used to generate this panel: DK5970 (*P_zpdG_-lacZ* ΔpBS32), DK5968 (*P_zpcJ_-lacZ* ΔpBS32), and DK5969 (*P_zpcX_-lacZ* ΔpBS32). Error bars are the standard deviation from three replicates. Data used to generate each panel are included in Table S5A to E. All panels use the same Y-axis.

To determine if and when SigN was expressed relative to MMC treatment, Western blot analysis was conducted. SigN protein was first detected 1 h after MMC treatment and continued to increase in abundance thereafter, whereas the vegetative sigma factor, SigA (σ^A^), was constitutive and constant (Fig. 1C). Loss of cell viability appeared to occur soon after MMC addition prior to observable SigN protein (e.g., 0.5 h. after addition [Fig. 1B]), and thus we inferred that SigN was expressed and active at levels below the limit of protein detection. To determine whether *sigN* transcription occurs soon after MMC treatment, the upstream intergenic region of *sigN* (P*_sigN_*) (Fig. 2A) was cloned upstream of the gene encoding β-galactosidase, *lacZ*, and inserted at an ectopic site in the chromosome (*aprE*::*P_sigN_*-*lacZ*). Expression from P*_sigN_* was low but increased 10-fold within an hour after MMC addition (*T*_1_), and the increase in expression was not dependent on the presence of pBS32 (Fig. 3A).

10.1128/mBio.01899-19.2 FIG S2 Rend-seq analysis of the *sigN* promoter region. The same data as in Fig. 2C re-presented after normalization per million reads that mapped to the plasmid. Download FIG S2, PDF file, 0.9 MB. Copyright © 2019 Burton et al. 2019 Burton et al. This content is distributed under the terms of the Creative Commons Attribution 4.0 International license.

10.1128/mBio.01899-19.5 TABLE S3 Rend-seq data table. List of primers used in the Rend-seq assay. Download Table S3, DOCX file, 0.01 MB. Copyright © 2019 Burton et al. 2019 Burton et al. This content is distributed under the terms of the Creative Commons Attribution 4.0 International license.

10.1128/mBio.01899-19.7 TABLE S5 β-Galactosidase activity in Miller units. Each table (A to F) lists the numerical values of each β-galactosidase assay performed in Miller units and identifies the corresponding figure. Download Table S5, DOCX file, 0.02 MB. Copyright © 2019 Burton et al. 2019 Burton et al. This content is distributed under the terms of the Creative Commons Attribution 4.0 International license.

To map the MMC response within the *sigN* promoter region, we split P*_sigN_* into two fragments, an upstream fragment called *P_sigN_^UP^* and a downstream fragment called *P_sigN_^DN^* (Fig. 2A). Both fragments were cloned upstream of *lacZ* and separately integrated into an ectopic site of the chromosome in a strain deleted for pBS32 and both chromosomal prophages, PBSX and SPβ (all strains are shown in Table 1). Basal expression from P*_sigN_^UP^* was at background levels but increased 100-fold when MMC was added (Fig. 3B). In contrast, expression from P*_sigN_^DN^* was at a constitutively low level and did not increase upon addition of MMC (Fig. 3B). We conclude that transcription of *sigN* is activated by MMC treatment, that the P*_sigN_^UP^* region contains an MMC-responsive promoter, and that MMC-dependent expression was controlled by a chromosomally encoded regulator as induction was not dependent on the presence of pBS32.

**TABLE 1:** Strains

Strain	Genotype (reference)
3610	Wild type
DS4203	*rpoC-hisX6Ω neo*(*kan*)
DK297	*ΔSPβ ΔPBSX* (17)
DK451	*ΔSPβ ΔPBSX Δ*pBS32 (17)
DK607	*ΔSPβ ΔPBSX ΔcomI*
DK1634	*ΔSPβ ΔPBSX ΔcomI amyE*::*P_hyspank_-zpdN^wkRBS^ spec* (17)
DK2862	*aprE*::*P_hag_-lacZ cat comI^Q12L^*
DK3287	*ΔSPβ ΔPBSX ΔcomI ΔsigN* (17)
DK4401	*amyE*::*P_hyspank_-sigN^wkRBS^ spec aprE*::*P_sigN_-lacZ cat*
DK4669	*amyE*::*P_hyspank_-sigN^wkRBS^ spec thrC*::*P_alfA_-lacZ mls*
DK4670	*amyE*::*P_hyspank_-sigN^wkRBS^ spec thrC*::*P_repN_-lacZ mls*
DK4671	*amyE*::*P_hyspank_-sigN^wkRBS^ spec thrC*::*P_comI_-lacZ mls*
DK4673	*amyE*::*P_hyspank_-sigN^wkRBS^ spec thrC*::*P_zpbK_-lacZ mls*
DK4725	*ΔSPβ ΔPBSX ΔcomI amyE*::*P_hyspank_-sigN^wkRBS^ spec thrC*::*P_comI_-lacZ mls*
DK4784	*ΔSPβ ΔPBSX aprE*::*P_sigN_-lacZ cat*
DK4943	*ΔSPβ ΔPBSX ΔcomI amyE*::*P_hyspank_-sigN^wkRBS^ spec aprE*::*P_sigN_-lacZ cat*
DK4948	*ΔSPβ ΔPBSX ΔcomI amyE*::*P_hyspank_-sigN^wkRBS^ spec thrC*::*P_alfA_-lacZ mls*
DK4949	*ΔSPβ ΔPBSX ΔcomI amyE*::*P_hyspank_-sigN^wkRBS^ spec thrC*::*P_repN_-lacZ mls*
DK4994	*ΔSPβ ΔPBSX ΔcomI amyE*::*P_hyspank_-sigN^wkRBS^ spec thrC*::*P_zpbK_-lacZ mls*
DK5066	*ΔSPβ ΔPBSX Δ*pBS32 *aprE*::*P_sigN_-lacZ cat*
DK5655	*ΔSPβ ΔPBSX ΔcomI amyE*::*P_hyspank_-sigN^wkRBS^ spec thrC*::*P_sigN_^UP^-lacZ mls*
DK5656	*ΔSPβ ΔPBSX ΔcomI amyE*::*P_hyspank_-sigN^wkRBS^ spec thrC*::*P_sigN_^DN^-lacZ mls*
DK5657	*ΔSPβ ΔPBSX Δ*pBS32 *amyE*::*P_hyspank_-sigN^wkRBS^ spec thrC*::*P_sigN_^UP^-lacZ mls*
DK5658	*ΔSPβ ΔPBSX Δ*pBS32 *amyE*::*P_hyspank_-sigN^wkRBS^ spec thrC*::*P_sigN_^DN^-lacZ mls*
DK5968	*ΔSPβ ΔPBSX Δ*pBS32 *amyE*::*P_hyspank_-sigN^wkRBS^ spec thrC*::*P_zpcJ_-lacZ mls*
DK5969	*ΔSPβ ΔPBSX Δ*pBS32 *amyE*::*P_hyspank_-sigN^wkRBS^ spec thrC*::*P_zpcX_-lacZ mls*
DK5970	*ΔSPβ ΔPBSX Δ*pBS32 *amyE*::*P_hyspank_-sigN^wkRBS^ spec thrC*::*P_zpdG_-lacZ mls*
DK7259	*ΔSPβ ΔPBSX Δ*pBS32 *lexA*::*mls aprE*::*P_sigN_^UP^-lacZ cat*
DK7260	*ΔSPβ ΔPBSX Δ*pBS32 *lexA*::*mls aprE*::*P_sigN_^DN^-lacZ cat*
DK7291	*ΔSPβ ΔPBSX Δ*pBS32 *aprE*::*P_sigN_^UP^-lacZ cat*
DK7292	*ΔSPβ ΔPBSX Δ*pBS32 *aprE*::*P_sigN_^DN^-lacZ cat*

One candidate for an MMC-responsive, chromosomally-encoded regulator is the transcriptional repressor protein LexA. LexA often binds sequences that overlap promoters to inhibit access of RNA polymerase holoenzyme (22, 23), and sequence analysis predicted a putative LexA-inverted repeat binding site located within the P*_sigN_^UP^* fragment (24, 25) (Fig. 2B). Moreover, target promoters are exposed and expression is derepressed when LexA undergoes autoproteolysis upon DNA damage like that caused by MMC (22, 23, 26). To determine if P*_sigN_^UP^* was LexA repressed, LexA was mutated in a background deleted for pBS32 and the two chromosomal prophages, PBSX and SPβ. Mutation of *lexA* dramatically increased expression from P*_sigN_^UP^* but not P*_sigN_^DN^* (Fig. 3C). We conclude that LexA either directly or indirectly inhibits expression of a promoter present in P*_sigN_^UP^* and that the MMC response was LexA mediated.

**FIG 4:**
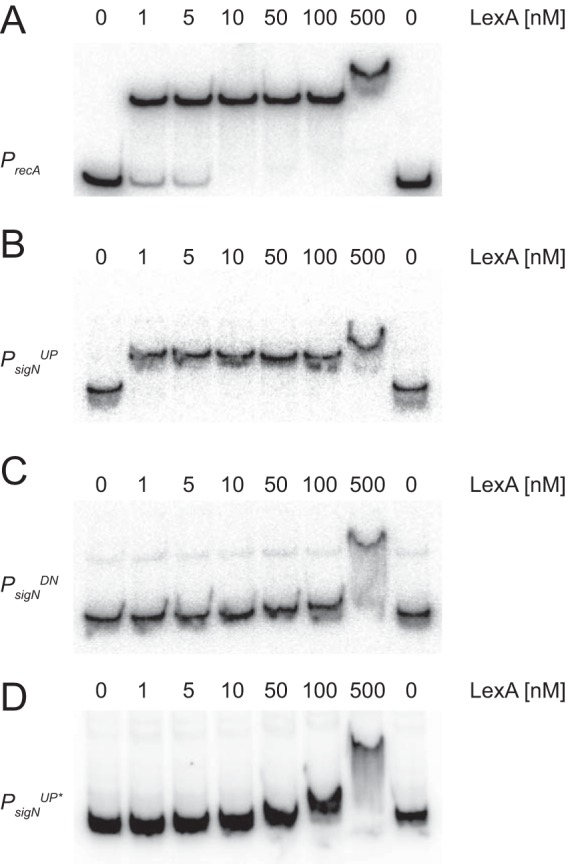
LexA binds to the *P_sigN_^UP^* promoter region. Electrophoretic mobility shift assays were performed with radiolabeled DNA of *P_recA_* (A), *P_sigN_^UP^* (B), *P_sigN_^DN^* (C), and P_sigN_^UP^* (D) mutated for the putative LexA binding site. Purified LexA protein was added to each reaction mixture at the indicated concentration.

One way that LexA might inhibit expression from P*_sigN_^UP^* is if it bound directly to the DNA. To determine whether LexA bound directly to the P*_sigN_^UP^* region, LexA was purified and added to various labeled DNA fragments in an electrophoretic mobility shift assay (EMSA). Consistent with direct, high-affinity binding, purified LexA caused an electrophoretic mobility shift in both the previously established target promoter P*_recA_* (23) (Fig. 4A) and the P*_sigN_^UP^* promoter region (Fig. 4B) at protein levels as low as 1 nM. LexA binding was specific as the affinity was reduced 500-fold for the P*_sigN_^DN^* promoter (Fig. 4C). Moreover, LexA binding was specific for the putative LexA inverted repeat sequence as mutation of the sequence (GAAC>TTAC) within P*_sigN_^UP^* reduced binding affinity 100-fold (Fig. 4D). We conclude that LexA binds to the P*_sigN_^UP^* promoter region and represses transcription.

**FIG 5:**
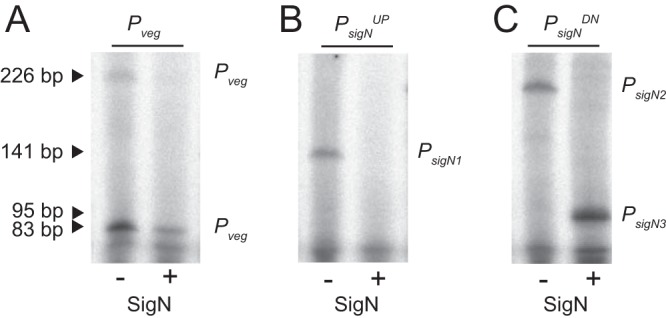
SigN is a sigma factor that drives transcription *in vitro*. *In vitro* transcription assays using *P_veg_* (left), *P_sigN_^UP^* (middle), and *P_sigN_^DN^* (right) promoter fragments in the presence (+) and absence (−) of a 5× molar ratio of SigN added to RNA polymerase holoenzyme purified from B. subtilis. The predicted transcriptional products resulting from *P_sigN1_*, *P_sigN2_*, and *P_sigN3_* are indicated. Two products were observed from *P_veg_* likely due to proper termination (short product) and terminator read-through (long product).

LexA often binds over the top of promoter elements (16), and sequence analysis suggested that the LexA inverted repeat in P*_sigN_^UP^* might rest immediately upstream of, and overlap, a putative SigA-dependent −35 promoter element (Fig. 2B). To determine whether P*_sigN_^UP^* contained a SigA-dependent promoter, RNA polymerase (RNAP) holoenzyme with SigA bound was purified from B. subtilis and used in *in vitro* transcription reactions (Fig. 5). Consistent with promoter activity, transcription product was observed when SigA-RNAP was mixed with either a known SigA-dependent promoter control, *P_veg_* (Fig. 5A, left lane), or the experimental P*_sigN_^UP^* (Fig. 5B, left lane). A transcription product was also observed when SigA-RNAP was mixed with the P*_sigN_^DN^* promoter fragment (Fig. 5C, left lane), consistent with low-level constitutive expression observed from reporters with that fragment (Fig. 3B). We conclude that there are two SigA-dependent promoters within the *P_sigN_* region, one within the P*_sigN_^UP^* fragment and one within the P*_sigN_^DN^* fragment.

To determine transcriptional start sites, Rend-seq (end-enriched transcriptome sequencing [RNA-seq]) analysis was performed for the entire B. subtilis transcriptome in the presence and absence of MMC treatment. Rend-seq achieves end enrichment by sparse fragmentation of extracted RNAs, which generates fragments containing original 5′ and 3′ ends, as well as a smaller amount of fragments containing internal ends (27, 28). Rend-seq indicated that expression of *sigN* was low in the absence of induction (Fig. 2C) but a 5′ end appeared within the P*_sigN_^UP^* region when MMC was added, the location of which was consistent with the SigA −10 promoter element predicted earlier (Fig. 2B) and supported later by *in vitro* transcription (Fig. 5B, left lane). We define the SigA-dependent promoter within P*_sigN_^UP^* as *P_sigN1_*. Rend-seq also indicated a weak but MMC-independent 5′ end within P*_sigN_^DN^* that was consistent with the *in vitro* transcription product originating from that fragment (Fig. 5C, left lane). Moreover, sequences consistent with SigA-dependent −35 and −10 promoter elements were identified upstream of the 5′ end within P*_sigN_^DN^* (Fig. 2B). We define the weak constitutive SigA-dependent promoter within P*_sigN_^DN^* as *P_sigN2_*. We conclude that there are two SigA-dependent promoters driving *sigN* expression and that *P_sigN1_* is both strong and LexA repressed.

### SigN is a sigma factor that activates its own expression.

Rend-seq analysis also indicated a second 5′ end within the P*_sigN_^DN^* fragment that would result in a slightly shorter transcript (Fig. 2C, peak marked *P_?_*). The shorter transcript could indicate either a highly specific RNA cleavage site in the 5′ upstream untranslated region of *sigN* or the presence of a third promoter with an individual start site. If there was a second promoter within P*_sigN_^DN^*, the promoter was presumably not dependent on SigA, as only one SigA-dependent transcript was observed from this fragment in *in vitro* transcription assays (Fig. 5C, left lane). One candidate for an alternative sigma factor that could drive expression of the third putative promoter is SigN itself. SigN is weakly homologous to extracytoplasmic function (ECF) sigma factors, and ECF sigma factors are often autoregulatory (29). Consistent with autoactivation, induction of SigN increased expression from P*_sigN_^DN^* 100-fold but did not increase expression from P*_sigN_^UP^* (Fig. 3D). We conclude that *sigN* expression is controlled by at least three promoters: a LexA-repressed SigA-dependent promoter, P*_sigN1_*; a weak constitutive SigA-dependent promoter, *P_sigN2_*; and a third promoter that was SigN dependent.

One way in which a promoter could be SigN dependent is if SigN is a bona fide sigma factor that directs its own transcription. To determine whether SigN had sigma factor activity, RNAP-SigA holoenzyme was purified from B. subtilis and purified SigN protein was added in 5-fold excess to *in vitro* transcription reaction mixtures (30–32). Addition of SigN reduced levels of the SigA-dependent *P_veg_*, *P_sigN1_*, and the *P_sigN2_*-derived transcripts, consistent with SigN competing with, and displacing, SigA from the RNA polymerase core (Fig. 5A and B, right lanes). Moreover, a new, shorter transcript appeared within *P_sigN_^DN^* that was SigN dependent (Fig. 5C, right lane). To map the location of the shorter transcript, Rend-seq was conducted on a strain that was artificially induced for SigN expression. Consistent with the *in vitro* transcription results, an intense SigN-dependent 5′ end was detected within the *P_sigN_^DN^* region which we infer is due to the presence of a promoter here called *P_sigN3_* (Fig. 2C). We note that the *P_sigN3_*-dependent transcript did not align with the original transcript peak from *P_?_* indicated by Rend-seq analysis, and thus, at least three and possibly more promoters may be present upstream of *sigN*. Moreover, both the *P_?_* and *P_sigN3_*-dependent peaks in the MMC-treated Rend-seq were abolished in *sigN* mutant cells, but only *P_sigN3_* was SigN stimulated (Fig. 2C). We conclude that SigN is a sigma factor that is necessary and sufficient for inducing expression from *P_sigN3_*.

Mapping of the Rend-seq transcriptional start site allowed prediction of the *P_sigN3_* promoter sequence (Fig. 2B). To determine the SigN regulon and consensus sequence, 5′-end Rend-seq products that increased at least 10-fold after artificial SigN induction were collected, and 40 bp of sequence upstream was compiled by MEME (33) (Fig. 2D and Table 2). A consensus sequence emerged that was consistent with the −35 and −10 regions predicted for *P_sigN3_* (Fig. 2B and D). Three separate promoter regions predicted to be regulated by SigN were cloned upstream of a promoterless *lacZ* gene and inserted at an ectopic site in the chromosome in a strain deleted for pBS32. In each case, the expression of the reporter was low during normal growth conditions but increased 100-fold when *sigN* was induced with isopropyl-β-d-thiogalactopyranoside (IPTG) (Fig. 3E). We conclude that SigN is a plasmid-encoded sigma factor that is necessary and sufficient for the expression of a regulon gene encoded on pBS32, and we infer that the expression of one or more genes within the SigN regulon is responsible for pBS32-mediated cell death.

**TABLE 2:** SigN-dependent promoters on pBS32

Promoter^*a*^	Sequence^*b*^	Operon^*c*^	Function^*d*^	Fold change^*e*^
*sigN*	TTTTCG**TTTACGTTT**CTATTTCTCTA**GATA**A**AA**TCATTAAG	*sigN*	Sigma factor	101
*zpaB*	TTCTC**ATTTACGTTT**TAGAAAGACTA**GATATAA**AGATTACG	*zpaB*	DNA gyrase	152.5
*zpaD*	TCTT**ATTTAC**A**T**AACTGGTTATGCCG**GATA**A**AA**GAAGATAG	*zpaDE*	Unknown	38
*zpbP*	CTACCA**ATTTACGTTT**CACCATTCTCA**GATATAA**ATATATT	*zpbP*	Unknown	158.4
*zpbS*	TTTTG**ATTTACG**AA**T**TCATATTCATA**GATATAA**GTATAAAA	*zpbS*	PG interaction	333.2
*zpbW*	TCC**ATT**A**A**TT**T**ACATATGGAAAATTACG**GATATAA**TCGTTA	*zpbW*	Regulator	178
*zpbY*	GAAAATCA**ATTTACGTTT**TCAAAGGCACA**GATATAA**TAACA	*zpbYZ zpcABCD*	Unknown	226
*zpcE*	TTTTTG**ATTTACGTTT**CTAAAACCCAA**GATATAA**AAGATAT	*zpcEFGH*	Nucleotide synth.	339.6
*zpcJ*	AATTA**ATTTACGTTT**TCCAAGAACCA**GATATAA**ATAAAAAG	*zpcJK*	Nucleotide synth.	245.9
*zpcL*	TTTTG**ATTTACGTTT**TTAATACTCCA**GATATAA**ATATTAAG	*zpcLM*	Nucleotide synth.	202.5
*zpcN*	TTATG**ATTTACGTTT**TTGTTTACCCA**GATA**A**AA**TAACAAAG	*zpcNOP*	Unknown	356.7
*zpcU*	GCTTG**ATTTACGTTT**TAAAAACCCCA**GATATAA**TAACGAAG	*zpcUV*	Exonuclease	263
*zpcX*	CATTA**ATTTACGTTT**TCGAATCACCA**GATATAA**ATAAAGAG	*zpcXYZ*	Nucleotide synth.	309.2
*zpdB*	TTTCA**ATTTACGTTT**TCGAATCACCA**GATATAA**ATACAAAG	*zpdBCDEF*	Nucleotide synth.	176.3
*zpdG*	ATCCA**ATTTACGTTT**TTGCCGGTCCA**GATATAA**ATACTTTG	*zpdG*	DNA Pol III	401
*zpdH1*	TCATA**ATTTACATTT**CTGTTATAACC**GATATAA**TACCCTCA	*zpdHIJKLM*	Nucleotide synth.	85
*zpdH2*	AAATG**ATTTACGTTT**TTCAATAACCA**GATATAA**ATATAAAG	*zpdHIJKLM*	Nucleotide synth.	297.7
*zpbQ*	TGTGG**TTTACGTTTT**AATAGAGCCA**GATATAA**TCATACCAA	*zpbQR*	Unknown	Predicted
*zpcR*	AA**TTTACGTTT**CAGGTGATCCA**GATATAA**TAACAAAAAATA	*zpcJKLMNOPQRSTUV*	Unknown	Predicted

a Promoter named by the first gene carried on the transcript predicted by Rend-seq analysis.

b Sequence of promoter obtained by taking the −40 to +1 position relative to the transcript predicted by Rend-seq analysis and used to generate Fig. 2D. Sequence matching the consensus is boldfaced.

c Operon obtained by the 3’ end of the transcript predicted by Rend-seq analysis.

d Function of gene/operon interpreted from BLAST results published in the work of Konkol et al. (7). PG, peptidoglycan; synth., synthesis; Pol, polymerase.

e Fold change calculated from Rend-seq values (SigN+++/WT). “Predicted” indicates that the Rend-seq did not indicate that the gene was a direct SigN target but that sequences upstream of the coding region consistent with the SigN consensus sequence were detected by bioinformatics.

## DISCUSSION

**FIG 6:**
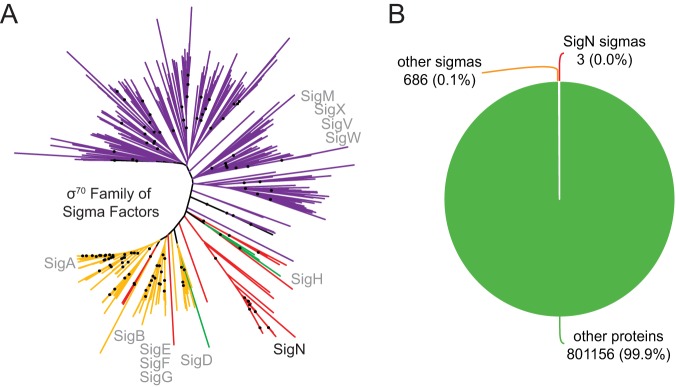
SigN distribution and phylogeny. (A) A phylogenetic tree indicating the relationships between members of the σ^70^ family of proteins. Sigma factors of the σ^70^ family were collected from 24 diverse bacterial genomes (Table S6). The colors represent which sigma factors were identified in the collection using the specified sigma factors from B. subtilis: gold, vegetative sigma, SigA (NCBI accession no. BAA25730.1); red, plasmid sigma, SigN (NCBI accession no. YP_008244202.1); green, stationary-phase sigma, SigH (NCBI accession no. QCJ19226.1); purple, extracytoplasmic (ECF) sigma, SigM (NCBI accession no. NP_388833.1). The relative location of SigN is labeled in black. The relative locations of other B. subtilis sigma factors are labeled in gray. Black dots indicate the locations of branches with bootstrap values greater than 70%. SigN homologs may represent a novel class of alternative σ^70^ sigma factors, but we cannot conclude this with certainty from the present analysis due to the absence of strong bootstrap values in the deepest branches of the tree. The tree can be visualized online at https://itol.embl.de/tree/1401827363250201560954077. (B) A pie chart indicating the frequency of sigma factors encoded on plasmids relative to the total number of plasmid-encoded proteins taken from a set of over 6,000 naturally occurring plasmids (34). The number of each class is given as an absolute value and as a percentage divided by all of the proteins encoded from the plasmid collection.

The pBS32-encoded protein SigN (formerly ZpdN) was shown to induce pBS32-mediated cell death and exhibit weak homology to sigma factors (17). Here, we show that SigN has sigma factor activity *in vitro*, and phylogenetic analysis suggests that SigN may represent a novel subclass of alternative sigma factors (Fig. 6A). Moreover, we used Rend-seq analysis to determine the regulon of genes under SigN control and the SigN consensus binding sequence (Fig. 2D and Table 2). Two additional SigN targets encoded on pBS32 were predicted using an unbiased search with the consensus sequence and may be subject to additional regulation (Table 2). A few seemingly unrelated chromosomal loci exceeded our threshold for determining SigN-induced targets, but subsequent bioinformatic analysis failed to find SigN promoters in the chromosome (see Table S4 in the supplemental material). Thus, the SigN regulon appears to be predominantly restricted to pBS32, at least in B. subtilis, and chromosomal effects could be either indirect or spurious.

10.1128/mBio.01899-19.6 TABLE S4 Rend-seq data of chromosomal genes. List of upregulated genes and corresponding fold change that was observed during SigN overexpression in the presence of pBS32. Download Table S4, DOCX file, 0.01 MB. Copyright © 2019 Burton et al. 2019 Burton et al. This content is distributed under the terms of the Creative Commons Attribution 4.0 International license.

10.1128/mBio.01899-19.8 TABLE S6 Genomes used for sigma 70 homolog sequence analysis. List of genomes used to obtain sigma 70 homologs for sequence analysis in Fig. 6B. Download Table S6, XLSX file, 0.01 MB. Copyright © 2019 Burton et al. 2019 Burton et al. This content is distributed under the terms of the Creative Commons Attribution 4.0 International license.

**FIG 7:**
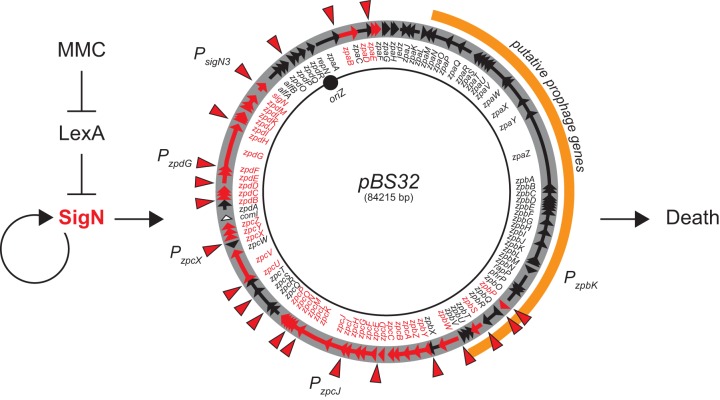
Model of pBS32-mediated cell death. MMC-mediated DNA damage causes LexA autoproteolysis and derepression of *sigN* expression. SigN is a sigma factor that directs RNA polymerase to increase its own expression (creating positive feedback) and the expression of a regulon of genes on pBS32. Activation of genes within the SigN regulon results in cell death. pBS32 is represented as a circle. Arrows within the circle indicate reading frames. The location of SigN-dependent promoters is indicated by red arrowheads. Reading frames and gene names that are expressed by SigN are indicated in red. T bars indicate inhibition, and arrows indicate activation. In black are the locations of specific promoters mentioned in the text.

Plasmid-encoded sigma factors are rare (34) (Fig. 6B), and we note that SigN homologs were also found in the chromosomes of some bacteria. Moreover, alternative sigma factors or analogs thereof are sometimes encoded within prophage elements (35–38), and pBS32 appears to encode a cryptic prophage. Whether pBS32 is, in its entirety, a P1-like plasmid prophage (39) or whether a phage secondarily lysogenized into a preexisting plasmid is unknown (17). Similar to, and perhaps consistent with, other lysogenic prophages in B. subtilis, DNA damage by MMC triggers hyperreplication of pBS32 and initiates pBS32-mediated cell death (16, 17, 40). Here, we show that MMC induces SigN-directed plasmid gene expression via the chromosomally encoded transcriptional repressor LexA (Fig. 7). LexA represses the *P_sigN1_* promoter, and MMC-mediated DNA damage promotes LexA autoproteolysis and derepression (26, 41–43). Derepression of *P_sigN1_* leads to vegetative SigA-dependent expression of SigN, and SigN accumulation locks the system into an activated state by positive feedback at the *P_sigN3_* promoter. SigN directs not only its own expression but an entire regulon on pBS32, which includes many genes homologous to those involved in nucleotide metabolism and DNA replication (7) (Table 2; Fig. 7). SigN activation causes pBS32 copy number to increase and either directly or indirectly promotes cell death (17).

How SigN promotes pBS32 hyperreplication is unknown. Replication of pBS32 requires the plasmid-encoded initiator protein RepN, and artificial overexpression of RepN was sufficient to increase plasmid copy number 100-fold (8). Thus, SigN could increase plasmid copy number by activating RepN expression, but *repN* did not increase in expression by Rend-seq analysis when SigN was artificially expressed. Moreover, the expression of a reporter in which the promoter of *repN* was fused to β-galactosidase also failed to increase when SigN was artificially induced (Fig. S1). We conclude that SigN does not directly activate *repN* transcription to promote plasmid hyperreplication. Moreover, pBS32 is normally a low-copy-number plasmid that requires active partitioning by the AlfAB system (10, 44, 45). Hyperreplication and/or imminent cell death may obviate active plasmid partitioning, and indeed, induction of SigN decreased expression from a reporter construct generated with the *P_alfAB_* promoter region (10, 44) (Fig. S1). Repression of *alfAB* appears to be indirect, however, as curing of pBS32 relieved SigN-dependent inhibition (Fig. S1). How SigN promotes hyperreplication remains unclear, but it may be an indirect effect caused by the mechanism of cell death.

10.1128/mBio.01899-19.1 FIG S1 SigN does not activate the replication initiator, partitioning system, competence inhibitor, or prophage structural genes. β-Galactosidase activity of strains containing either *P_sigN_-lacZ*, *P_repN_-lacZ* (promoter of the replication initiator protein RepN), *P_alfA_-lacZ* (promoter of the partitioning system AlfAB), *P_comI_-lacZ* (promoter of the competence inhibitor ComI), or *P_zpbK_-lacZ* (promoter of the long prophage structural gene operon) in the absence (open bars) or presence (closed bars) of IPTG. Reporter expression was measured in cells containing (left panel) or lacking (right panel) pBS32. The following strains were used to generate this panel: DK4401 (*P_sigN_-lacZ Δ*pBS32), DK4670 (*P_repN_-lacZ Δ*pBS32), DK4669 (*P_alfA_-lacZ Δ*pBS32), DK4671 (*P_comI_-lacZ Δ*pBS32), DK4673 (*P_zpbK_-lacZ Δ*pBS32), DK4943 (*P_sigNI_-lacZ*), DK4949 (*P_repN_-lacZ*), DK4948 (*P_alfA_-lacZ*), DK4725 (*P_comI_-lacZ*), and DK4994 (*P_zpbK_-lacZ*). Error bars are the standard deviation from three replicates. Data used to generate each panel are included in Table S5F. Download FIG S1, PDF file, 0.05 MB. Copyright © 2019 Burton et al. 2019 Burton et al. This content is distributed under the terms of the Creative Commons Attribution 4.0 International license.

How SigN and pBS32 promote cell death is complex. In the presence of pBS32, cells activated for SigN decrease in viability 1,000-fold even as cells increase in optical density for three generations. Thus, toxicity likely is not due to direct inhibition of a(n) essential component(s), and instead, something essential appears to be depleted and not replaced. Hyperreplication of the plasmid may deplete nucleotide pools, but at present we cannot determine whether hyperreplication and death are linked or separate phenotypes. Death might be mediated by the prophage structural and lytic genes, and we note that a SigN homolog is encoded by VPE25, a phage that infects *Enterococcus* (46). SigN-dependent promoters, however, appear to be largely excluded from the prophage region (Fig. 7), and while prophage gene expression increased in Rend-seq, the increase might have been due to the increase in plasmid copy number alone. Moreover, expression of a reporter in which the *P_zpbK_* promoter of the phage structural operon (as predicted by Rend-seq) was fused to β-galactosidase was found to be pBS32 dependent but SigN insensitive (Fig. S1). Finally, large deletions of the prophage structural genes did not abolish pBS32-mediated cell death (17). Thus, SigN-mediated cell death may be separate from prophage gene expression, at least in B. subtilis.

Alternative sigma factors may be activated to promote gene expression for the purpose of adapting to environment stress (47, 48). Here, we show that SigN is a sigma that is derepressed in response to DNA damage and in turn leads to cell death. Why a plasmid encodes a sigma factor that kills its host is unclear, unless perhaps the entire plasmid is a defective prophage and SigN, at one time, promoted lytic conversion (17). The pBS32 plasmid also encodes ComI, an inhibitor of horizontal transfer by natural competence (7). Perhaps, ComI was needed to suppress DNA uptake because the import of single-stranded DNA creates a low-level DNA damage response that could have derepressed LexA and spuriously initiated SigN-mediated cell death during transformation (40, 49). Alternatively, SigN might be activated independently of the DNA damage response. We note that there is a third weak but constitutive promoter (*P_sigN2_*) that also drives expression of SigN. The function of *P_sigN2_* and the reason that *P_sigN2_* is insufficient to promote SigN-mediated cell death are unknown. We speculate, however, that *P_sigN2_* may either provide for additional environmental regulation on SigN or merely be a vestige of former regulation. Ultimately, why B. subtilis retains a potentially lethal plasmid and a sigma factor that promotes cell death is unknown.

## MATERIALS AND METHODS

### Strains and growth conditions.

B. subtilis strains were grown in lysogeny broth (LB) (10 g tryptone, 5 g yeast extract, 5 g NaCl per liter) or on LB plates fortified with 1.5% Bacto agar at 37°C. When appropriate, antibiotics were used at the following concentrations: 5 μg/ml kanamycin, 100 μg/ml spectinomycin, 5 μg/ml chloramphenicol, 10 μg/ml tetracycline, and 1 μg/ml erythromycin with 25 μg/ml lincomycin (*mls*). Mitomycin C (MMC; DOT Scientific) was added to the medium at the indicated concentration when appropriate. Isopropyl-β-d-thiogalactopyranoside (IPTG; Sigma) was added to the medium as needed at the indicated concentration.

### Strain construction.

All constructs were first introduced into the domesticated strain PY79 or into the pBS32 cured strain (DS2569) by natural competence and then transferred into the 3610 background using SPP1-mediated generalized phage transduction (49). Strains were also produced by transforming directly into the competent derivatives of 3610: DK607 (Δ*comI*) or DK1042 (Q-to-L change at position 12 encoded by *comI*). All strains used in this study are listed in Table 1. All plasmids used in this study are listed in Table S1 in the supplemental material. All primers used in this study are listed in Table S2.

10.1128/mBio.01899-19.3 TABLE S1 Plasmids. List of plasmids used in this study. Download Table S1, DOCX file, 0.01 MB. Copyright © 2019 Burton et al. 2019 Burton et al. This content is distributed under the terms of the Creative Commons Attribution 4.0 International license.

10.1128/mBio.01899-19.4 TABLE S2 Primers. List of primers used in this study. Download Table S2, DOCX file, 0.01 MB. Copyright © 2019 Burton et al. 2019 Burton et al. This content is distributed under the terms of the Creative Commons Attribution 4.0 International license.

### (i) *lacZ* reporter constructs.

To generate the β-galactosidase (*lacZ*) reporter construct *aprE::P_sigN_-lacZ cat*, PCR was utilized to amplify the promoter region of *sigN* using the primer set 4500/4528 from B. subtilis 3610 chromosomal DNA and primer set 4438/4501 was used to amplify the *aprE* up region and Cat^r^ from DK2862, while primer set 4527/4441 was used to amplify the *aprE* down region and *lacZ* from DK2862. These DNA fragments were ligated together in a Gibson isothermal assembly (ITA) reaction (see below) for 1 h at 60°C. Cementing PCR was performed using primer set 4438/4441 and the product was cleaned up using a QIAquick PCR purification kit (Qiagen). The resulting DNA fragment was transformed into DK1042.

To generate the *P_sigN_^UP^* and *P_sigN_^DN^* β-galactosidase reporter constructs at *thrC*, the promoter region of *sigN* was amplified via PCR with the primer set 6089/6090 for *P_sigN_^UP^* and 6087/6088 for *P_sigN_^DN^* from B. subtilis 3610 chromosomal DNA. Each PCR product was digested with EcoRI and BamHI and cloned independently into the EcoRI and BamHI sites of plasmid pDG1663, which carries an erythromycin resistance marker and a polylinker upstream of the *lacZ* gene between the two arms of the *thrC* gene to create pATB9 and pATB10, respectively. These plasmids were transformed into DK1042.

To generate the *P_sigN_^UP^* and *P_sigN_^DN^* β-galactosidase reporter constructs at *aprE*, the first half of the promoter was PCR amplified using primers 4500 and 4707 from B. subtilis 3610 chromosomal DNA. The second half of the promoter was amplified using primer set 4708/4528 from B. subtilis 3610 chromosomal DNA. For *P_sigN_^UP^*, the flanking regions of AprE were amplified as described above. For *P_sigN_^DN^*, the flanking regions were amplified with the following primer sets: *aprE* up region and Cat^r^ (4438/4498) and *aprE* down region and *lacZ* (4527/4441) from DK2862. Each promoter region was fused to the respective flanking arms of the *aprE* region using Gibson ITA as described above. The fused and amplified fragments were transformed into DK1042.

To generate the *P_zpcJ_*, *P_zpcX_*, and *P_zpdG_* β-galactosidase reporter constructs, primer sets were used in the following order to amplify each promoter region: 6276/6277 (*P_zpcJ_*), 6278/6279 (*P_zpcX_*), and 6280/6281 (*P_zpdG_*). Each promoter region was digested with EcoRI and BamHI and subsequently cloned independently into the EcoRI and BamHI sites of plasmid pDG1663, which carries an erythromycin resistance marker and a polylinker upstream of the *lacZ* gene between the two arms of the *thrC* gene to create pATB12, pATB13, and pATB14, respectively. These plasmids were transformed into DK1042.

To generate the *P_repN_*, *P_alfA_*, *P_comI_*, and *P_zpbK_* β-galactosidase reporter constructs, primer sets were used in the following order to amplify each promoter region: 5050/5051 (*P_repN_*), 5048/5049 (*P_alfA_*), 5052/5053 (*P_comI_*), and 5122/5123 (*P_zpbK_*). Each promoter region was digested with EcoRI and BamHI and subsequently cloned independently into the EcoRI and BamHI sites of plasmid pDG1663, which carries an erythromycin resistance marker and a polylinker upstream of the *lacZ* gene between the two arms of the *thrC* gene to create pDP477, pDP476, pDP478, and pDP480, respectively. These plasmids were transformed into DK1042.

### (ii) *lexA*::*mls*.

The *lexA*::*mls* insertion deletion allele was generated using a modified “Gibson” isothermal assembly protocol (50). Briefly, the region upstream of *lexA* was PCR amplified using the primer pair 5661/5662 and the region downstream of *lexA* was PCR amplified using the primer pair 5663/5664. To amplify the *erm* resistance gene, pAH52 plasmid DNA was used in a PCR with the universal primers 3250/23251. Fragments were added in equimolar amounts to the Gibson ITA reaction mixture, and the reaction was performed as explained above. The mixture from the completed reaction was then PCR amplified using primers 5661/5664 to amplify the assembled product. The product was transformed into DK1042.

### (iii) Isothermal assembly reaction buffer (5×).

A mixture containing 500 mM Tris-HCl (pH 7.5), 50 mM MgCl_2_, 50 mM dithiothreitol (DTT) (Bio-Rad), 31.25 mM polyethylene glycol (PEG) 8000 (Fisher Scientific), 5.02 mM NAD (Sigma-Aldrich), and 1 mM (each) deoxynucleoside triphosphate (dNTP) (New England BioLabs) was aliquoted and stored at −80°C. An assembly master mixture was made by combining prepared 5× isothermal assembly reaction buffer (131 mM Tris-HCl, 13.1 mM MgCl_2_, 13.1 mM DTT, 8.21 mM PEG 8000, 1.32 mM NAD, and 0.26 mM [each] dNTP) with Phusion DNA polymerase (New England BioLabs) (0.033 units/μl), T5 exonuclease diluted 1:5 with 5× reaction buffer (New England BioLabs) (0.01 units/μl), *Taq* DNA ligase (New England BioLabs) (5,328 units/μl), and additional dNTPs (267 μM). The master mix was aliquoted as 15 μl and stored at −80°C.

### SPP1 phage transduction.

To an 0.2-ml dense culture grown in TY broth (LB supplemented with 10 mM MgSO_4_ and 100 μM MnSO_4_ after autoclaving), serial dilutions of SPP1 phage stock were added. This mixture was allowed to statically incubate at 37°C for 15 min. A 3-ml volume of TYSA (molten TY with 0.5% agar) was added to each mixture and poured on top of fresh TY plates. The plates were incubated at 37°C overnight. Plates on which plaques formed had the top agar harvested by scraping into a 50-ml conical tube. To release the phage, the tube was vortexed for 20 s and centrifuged at 5,000 × *g* for 10 min. The supernatant was passed through an 0.45-μm syringe filter and stored at 4°C.

Recipient cells were grown in 2 ml of TY broth at 37°C until stationary phase was reached. A 5-μl volume of SPP1 donor phage stock was added to 1 ml of cells, and 9 ml of TY broth was added to this mixture. The transduction mixture was allowed to stand statically at room temperature for 30 min. After incubation, the mixture was centrifuged at 5,000 × *g* for 10 min, the supernatant was discarded, and the pellet was resuspended in the volume left. One hundred to 200 μl of the cell suspension was plated on TY fortified with 1.5% agar, 10 mM sodium citrate, and the appropriate antibiotic for selection.

### Protein purification.

To create the SUMO-SigN fusion protein expression vector, the coding sequence of SigN was amplified from 3610 genomic DNA with primers that also introduced a SapI site at the 5′ end and a BamHI site at the 3′ end. This fragment was ligated into the SapI and BamHI sites of pTB146 to create pBM05.

To purify SigN, pBM05 was expressed in Rosetta Gami II cells and grown at 37°C until mid-log phase (∼0.5 OD_600_). IPTG was added to the cells to induce protein expression, and cells were allowed to grow overnight at 16°C. Cells were harvested by centrifugation, washed, and emulsified with EmulsiFlex-C3 (Avestin). Lysed cells were ultracentrifuged at 14,000 × *g* for 30 min at 4°C.The supernatant was mixed with Ni^2+^-NTA His·Bind resin (EMD Millipore) equilibrated with lysis/binding buffer (50 mM Na_2_HPO_4_, 300 mM NaCl, 10 mM Imidazole, final pH 7.5) and allowed to incubate overnight at 4°C. The bead-lysate mixture was allowed to pack in a 1-cm separation column (Bio-Rad) and washed with wash buffer (50 mM Na_2_HPO_4_, 300 mM NaCl, 30 mM Imidazole, final pH 7.5). His-SUMO-SigN bound to the resin and was eluted using a stepwise elution of wash buffer with 50 to 500 mM imidazole and 10% glycerol to a final pH of 7.5. Eluates were separated by SDS-PAGE and stained with Coomassie brilliant blue to verify purification. Purified His-SUMO-SigN was combined with ubiquitin ligase (protease) and cleavage buffer and incubated at room temperature for 4 h to cleave the SUMO tag from the SigN protein (51). The cleavage reaction mixture was combined with Ni^2+^-NTA His·Bind resin, incubated for 1 h at 4°C, and centrifuged to pellet the resin. Supernatant was removed and dialyzed into lysis/binding buffer without the imidazole (50 mM Na_2_HPO_4_, 300 mM NaCl, 20% glycerol, final pH 7.5). Removal of the tag was confirmed by SDS-PAGE and staining with Coomassie brilliant blue.

To purify RNA polymerase, LB supplemented with kanamycin (5 μg/ml) was inoculated with an overnight culture of DK4203, which has the *rpoC-hisX6* construct integrated into the native site of *rpoC*. The cells were grown at 37°C until they hit mid-log phase (∼0.5 OD_600_) and were harvested via centrifugation. The collected cells were washed with buffer I (10 mM Tris-HCl [pH 8.0], 0.1 M KCl, 1 mM β-mercaptoethanol, 10% [vol/vol] glycerol) twice, resuspended in buffer G (10 mM Tris-HCl [pH 8.0], 20% [vol/vol] glycerol, 10 mM imidazole, 0.5 mg/ml lysozyme), and emulsified with EmulsiFlex-C3 (Avestin). The extracts were centrifuged for 30 min at 28,000 × *g* twice. The supernatant was mixed with Ni^2+^-NTA His·Bind resin (EMD Millipore) equilibrated with buffer G and allowed to go overnight at 4°C. The resin was collected by centrifugation and washed with buffer G. Buffer E (10 mM Tris-HCl [pH 8.0], 20% [vol/vol] glycerol, 500 mM imidazole) was used to elute the proteins associated with the resin and dialyzed in TGED buffer (10 mM Tris-HCl [pH 8.0], 1 mM EDTA, 0.3 mM DTT, 20% [vol/vol] glycerol).

To create the SUMO-LexA fusion protein expression vector, the coding sequence of LexA was amplified from 3610 genomic DNA with primers that also introduced a SapI site at the 5′ end and a BamHI site at the 3′ end. This fragment was ligated into the SapI and BamHI sites of pTB146 to create pATB11.

For the purification of LexA, pATB11 was expressed in Rosetta Gami II cells and grown at 37°C until mid-log phase (∼0.5 OD_600_). Cells were treated the same as in the protein purification procedure for SigN (above).

### SigN antibody purification.

One milligram of purified SigN protein was sent to Cocalico Biologicals for serial injection into a rabbit host for antibody generation. Anti-SigN serum was mixed with SigN-conjugated Affigel-10 beads and incubated overnight at 4°C. Beads were packed onto a 1-cm column (Bio-Rad), washed with 100 mM glycine (pH 2.5) to release the antibody, and neutralized immediately with 2 M Tris base. The antibody was verified using SDS-PAGE and stained with Coomassie brilliant blue. Purified anti-SigN antibody was dialyzed into 1× phosphate-buffered saline (PBS) with 50% glycerol and stored at −20°C.

### Western blotting.

B. subtilis strains were grown in LB and treated with mitomycin C (final concentration 0.3 μg/ml) as reported in the work of Myagmarjav et al. (17). Cells were harvested by centrifugation at the different time points after treatment. Cells were resuspended to an OD_600_ of 10 in lysis buffer (20 mM Tris-HCl [pH 7.0], 10 mM EDTA, 1 mg/ml lysozyme, 10 μg/ml DNase I, 100 μg/ml RNase I, 1 mM phenylmethylsulfonyl fluoride [PMSF]) and incubated for 1 h at 37°C. Twenty microliters of lysate was mixed with 4 μl 6× SDS loading dye. Samples were separated by 12% sodium dodecyl sulfate-polyacrylamide gel electrophoresis (SDS-PAGE). The proteins were electroblotted onto nitrocellulose and developed with a primary antibody used at a 1:5,000 dilution of anti-SigN, a 1:80,000 dilution of anti-SigA, and a 1:10,000 dilution secondary antibody (horseradish peroxidase-conjugated goat anti-rabbit immunoglobulin G). The immunoblot was developed using the Immun-Star horseradish peroxidase (HRP) developer kit (Bio-Rad).

### β-Galactosidase assay.

Biological replicates of B. subtilis strains were grown in LB and treated with mitomycin C to a final concentration of 0.3 μg/ml. Cells were allowed to grow, and 1 ml was harvested by centrifugation at the different time points indicated after treatment. When IPTG (final concentration, 1 mM) was used, cells grew to an OD_600_ of 0.6 and 1 ml was harvested. Cells were resuspended in 1 ml of Z buffer (40 mM NaH_2_PO_4_, 60 mM Na_2_HPO_4_, 1 mM MgSO_4_, 10 mM KCl, and 38 mM β-mercaptoethanol) with 0.2 mg/ml of lysozyme and incubated at 30°C for 15 min. Each sample was diluted accordingly with Z buffer to 500 μl. The reaction was started with 100 μl of 4 mg/ml *O*-nitrophenyl-β-d-galactopyranoside (in Z buffer) and stopped with 1 M Na_2_CO_3_ (250 μl). The OD_420_ of each reaction mixture was noted, and the β-galactosidase specific activity was calculated using the equation [OD_420_/(time × OD_600_)] × dilution factor × 1,000.

### Collection of cells for Rend-seq.

Overnight cultures were back diluted 1:100 in LB and grown at 37°C with shaking. When the cultures reached an OD_600_ of 0.1, they were treated with either 1 μg/ml MMC (DK297 and DK3287) or 1 mM IPTG (DK1634). The *zpdN* overexpression strain was harvested 1 h after induction by IPTG. Cells treated with MMC were collected after 2 h. After treatment, 10 ml of each culture was mixed with 10 ml of ice-cold methanol and spun down at 3,220 × *g* at 4°C for 10 min. Supernatant was discarded, and cell pellets were frozen in liquid nitrogen and stored at −80°C. For RNA extraction, the thawed pellets were resuspended in 1 ml of TRIzol reagent (Thermo Fisher, Waltham, MA) and added to FastPrep Lysis Matrix B 2-ml tubes with beads (MP Biomedicals). Cells were disrupted in a Bead Ruptor 24 (Omni International, Kennesaw, GA) twice for 40 s at 6.0 M/s. Two hundred microliters of chloroform was and were kept at room temperature for 2 min after vigorous vortexing. The mixture was spun down at 18,200 × *g* for 30 min at 4°C. The aqueous phase (∼600 μl) was precipitated with 900 μl of isopropanol for 10 min at room temperature. The RNA pellet was collected and washed with 80% ethanol.

### Rend-seq library preparation.

RNA was prepared for Rend-seq as described in detail in the work of Lalanne et al. (27) and DeLoughery et al. (28). In brief, 5 to 10 μg RNA was DNase treated (Qiagen) and rRNA was depleted (MICROBExpress; Thermo Fisher). rRNA-depleted RNA was fragmented by first heating the sample to 95°C for 2 min and adding RNA fragmentation buffer (1×; Thermo Fisher) for 30 s at 95°C and quenched by addition of RNA fragmentation stop buffer (Thermo Fisher). RNA fragments between 20 and 40 bp were isolated by size excision from a denaturing polyacrylamide gel (15%, Tris-borate-EDTA [TBE]–urea, 65 min, 200 V; Thermo Fisher). RNA fragments were dephosphorylated using T4 polynucleotide kinase (New England BioLabs, Ipswich, MA), precipitated, and ligated to 5′-adenylated and 3′-end-blocked linker 1 (IDT; 5 μM) using T4 RNA ligase 2, truncated K227Q. The ligation was carried out at 25°C for 2.5 h using <5 pmol of dephosphorylated RNA in the presence of 25% PEG 8000 (Thermo Fisher). cDNA was prepared by reverse transcription of ligated RNA using Superscript III (Thermo Fisher) at 50°C for 30 min. with primer oCJ485 (IDT, Coralville, IA), and the RNA was hydrolyzed. cDNA was isolated by PAGE size excision (10% TBE-urea, 200 V, 80 min; Thermo Fisher). Single-stranded cDNAs were circularized using CircLigase (Illumina, San Diego, CA) at 60°C for 2 h. Circularized cDNA was the template for PCR amplification using Phusion DNA polymerase (New England BioLabs) with Illumina sequencing primers, primer o231 (IDT), and barcoded indexing primers (IDT). After 6 to 10 rounds of PCR amplification, the product was selected by size from a nondenaturing PAGE gel (8% TB (tris-borate), 45 min, 180 V; Life Technologies). For data set names and barcode information, see Table S3 in the supplemental material.

### RNA sequencing and data analysis.

Sequencing was performed on an Illumina HiSeq 2000 sequencer. The 3′ linker sequences were stripped. Bowtie v. 1.2.1.1 (options -v 1 -k 1) was used for sequence alignment to the reference genome NC 000964.3 (B. subtilis chromosome) and KF365913.1 (B. subtilis plasmid pBS32) obtained from the NCBI Reference Sequence Bank. To deal with nontemplate addition during reverse transcription, reads with a mismatch at their 5′ end had their 5′ end reassigned to the immediate next downstream position. The 5′ and 3′ ends of mapped reads between 15 and 45 nucleotides (nt) in size were counted separately at genomic positions to produce wig files. The wig files were normalized per million non-rRNA and non-tRNA reads for each sample. Shadows were removed from wig files first by identifying the position of peaks and then by reducing the other end of the aligned reads by the peak’s enrichment factor to produce the final normalized and shadow-removed wig files. Gene regions were plotted in Matlab.

### Electromobility shift assays.

To perform electromobility shift assays, LexA was purified from Escherichia coli as outlined above. The control promoter, *P_recA_*, was amplified using the primer set 6252/6253, *P_sigN_^UP^* was amplified using the primer set 6089/6090, *P_sigN_^DN^* was amplified using the primer set 6087/6088, and P_sigN_^UP^* (LexA site scrambled) was amplified using the primer sets 6089/6284 and 6090/6283 from B. subtilis 3610 genomic DNA. The P_sigN_^UP^* fragments were ligated using Gibson ITA as outlined above. All fragments were cleaned up using the QIAquick PCR purification kit (Qiagen). Each DNA probe was end labeled with [γ-^32^P]ATP with T4 polynucleotide kinase (PNK) (New England BioLabs). Excess nucleotide was removed using G-50 microcolumns (GE Life Technologies). DNA binding reaction mixtures contained 4 nM DNA probe and a specific concentration of purified LexA protein (either 1, 5, 10, 50, 100, or 500 nM). Reactions were carried out in binding buffer (100 mM HEPES, pH 7.5, 100 mM Tris-HCl, 50% glycerol, 500 mM NaCl, 10 mM EDTA, 10 mM DTT) supplemented with 100 μg/ml bovine serum albumin (BSA) and 10 ng/μl poly(dI-dC). All reaction mixtures were incubated for 45 min at room temperature. Protein-DNA complexes were resolved on a 6% TGE (tris-glycine EDTA) polyacrylamide gel. Gels were dried at 80°C for 90 min and exposed to a storage phosphor screen overnight. Gels were imaged with a Typhoon 9500 imager (GE Life Sciences).

### *In vitro* transcription.

DNA template (50 ng) was mixed with either RNAP only (250 nM) or RNAP plus SigN (1,000 nM) per reaction. Each reaction mixture was incubated for 15 min at 37°C in a 25-μl total reaction volume including the transcription buffer (18 mM Tris-HCl [pH 8.0], 10 mM MgCl_2_, 30 mM NaCl, 1 mM DTT, 250 μM GTP, 100 μM ATP, 100 μM CTP, 5 μM UTP, and ∼2 μCi [α-^32^P]UTP) to produce multiple-round transcription. To stop the reaction, 25 μl of 2× Stop/Gel loading solution (7 M urea, 10 mM EDTA, 1% SDS, 2× TBE, 0.05% bromophenol blue) was used. Samples were run on a 5% denaturing acrylamide gel (5% acrylamide [19:1 acryl:bis], 7 M urea, 1× TBE) for 3 h at 200 V. Gels were imaged with a Typhoon 9500 imager (GE Life Sciences).

### Phylogenetic tree creation.

Sigma homologs to SigA, SigH, and SigM were identified in the genomes of 24 model organisms (listed in Table S6) using BLAST+ version 2.2.31 and an E value threshold of 1E−2 (52) and compared to all SigN homologs found in the database. The sequences were aligned using the default parameters of Muscle v3.8.31. The resulting alignment was used to generate a phylogenetic tree with RAxML version 8.1.3 set to perform 100 rapid bootstraps and subsequent maximum likelihood search using the GAMMA model of rate heterogeneity and the JTT substitution model (53). The data are presented using the Interactive Tree of Life visualization software (54).

### Data and software availability.

Ribosome profiling and RNA sequencing are available at the Gene Expression Omnibus under accession number GSE134424. Data were analyzed using custom Matlab scripts which are available upon request.
