# Supplementary figures and images for: Transcriptional Regulation and Mechanism of SigN (ZpdN), a pBS32-Encoded Sigma Factor in Bacillus subtilis
Source: mBio. 2019 Sep 17;10(5):e01899-19. doi: 10.1128/mBio.01899-19 (PMC6751061; doi:10.1128/mBio.01899-19)

Supplemental Figure S2

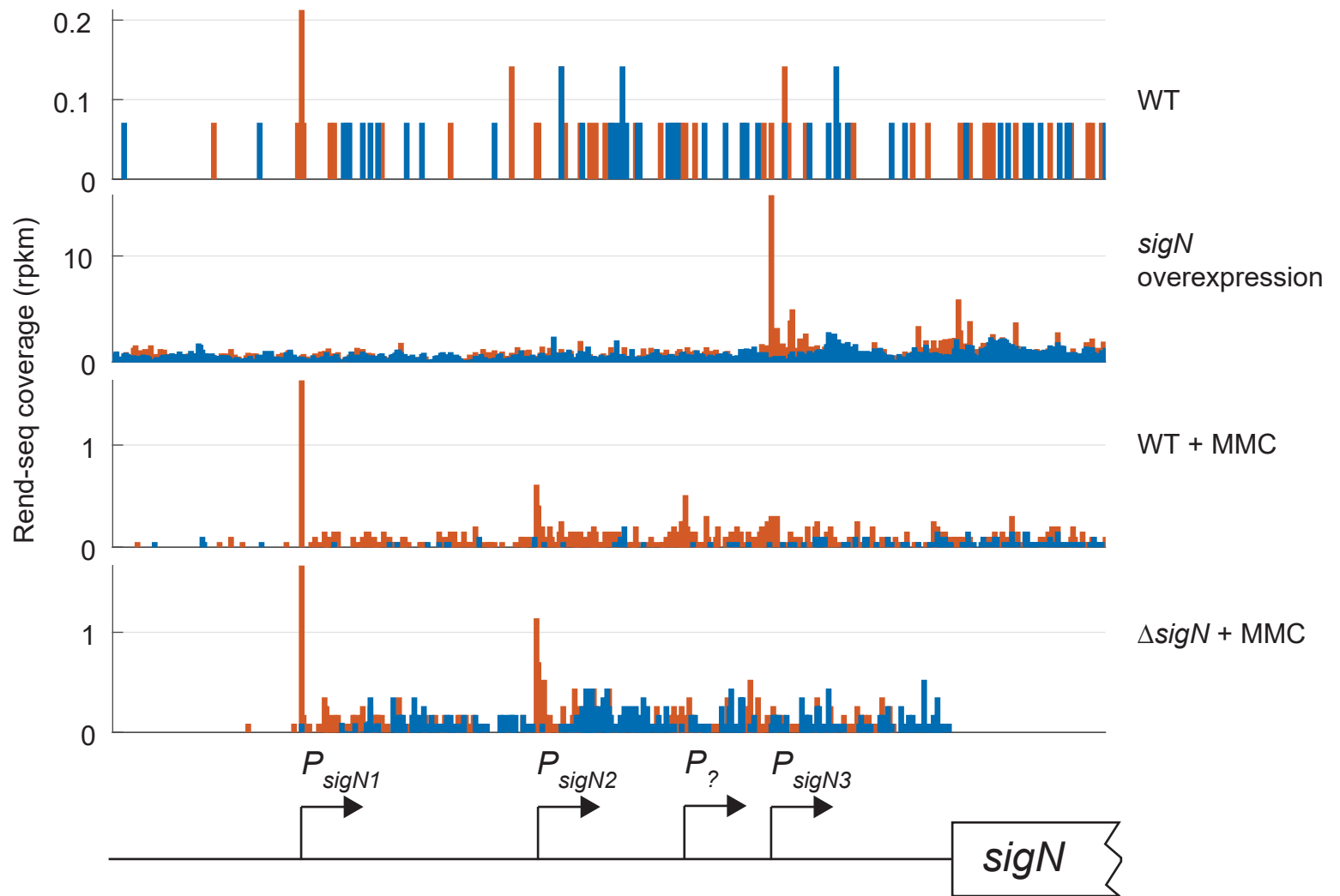

Supplement: FIG S2 [file mBio.01899-19-sf002.pdf]

# Supplemental Figure 1

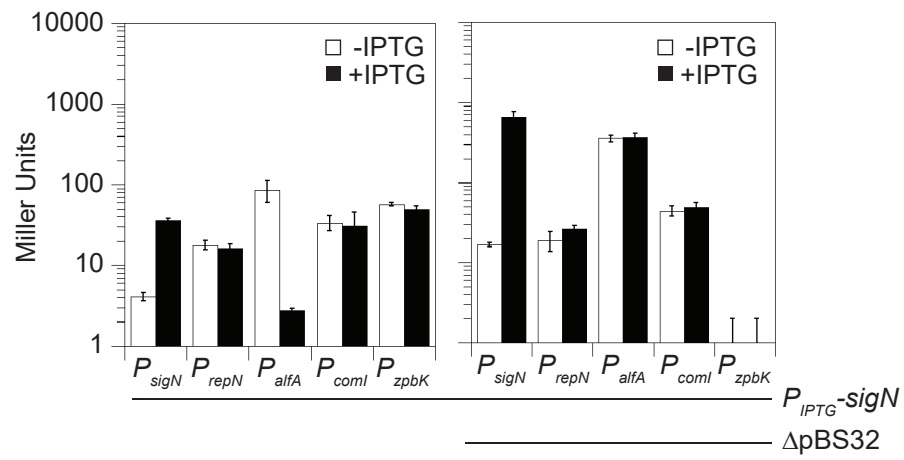

Supplement: FIG S1 [file mBio.01899-19-sf001.pdf]
