# Supplementary material for: Transcriptional Regulation and Mechanism of SigN (ZpdN), a pBS32-Encoded Sigma Factor in Bacillus subtilis
Source: mBio. 2019 Sep 17;10(5):e01899-19. doi: 10.1128/mBio.01899-19 (PMC6751061; doi:10.1128/mBio.01899-19)
Supplement: TABLE S3 [file mBio.01899-19-st003.docx]

**Table S3: Rend-Seq Data Table**

| **Barcode Label** |  |
| --- | --- |
| D17-9147 | ADAB1 (ATCACG) WT untreated |
| D17-9148 | ADAB2 (CGATGT) *sigN* over expression 1 hr post induction |
| D17-9149 | ADAB3 (TTAGGC) WT 2 hrs MMC treatment |
| D17-9150 | ADAB4 (TGACCA) *∆sigN* 2 hrs MMC treatment |

| Sequencing Library Prep | |
| --- | --- |
| indexing primer | Aatgatacggcgaccaccgagatctacacgatcggaagagcacacgtctgaactccagt  cacNNNNNNacactctttccctacac |
| oCJ485 | /5Phos/AGATCGGAAGAGCGTCGTGTAGGGAAAGAGTGT/iSp18/CAAG  CAGAAGACGGCATACGAGATATTGATGGTGCCTACAG |
| linker 1 | rAppCTGTAGGCACCATCAATÐNH2 |
