# Supplementary material for: Transcriptional Regulation and Mechanism of SigN (ZpdN), a pBS32-Encoded Sigma Factor in Bacillus subtilis
Source: mBio. 2019 Sep 17;10(5):e01899-19. doi: 10.1128/mBio.01899-19 (PMC6751061; doi:10.1128/mBio.01899-19)
Supplement: TABLE S5 [file mBio.01899-19-st005.docx]

**Table S5A: β-galactosidase activity in Miller units for Figure 3A.**

| Genotype | Strain | 0 h | 0.5 h | 1 h | 1.5 h | 2 h |
| --- | --- | --- | --- | --- | --- | --- |
| *P_sigN_-lacZ* | DK4784 | 4 ± 1 | 14 ± 7 | 40 ± 4 | 44 ± 13 | 53 ± 22 |
| *P_sigN_-lacZ*  ΔpBS32 | DK5066 | 6 ± 2 | 19 ± 9 | 39 ± 8 | 43 ± 10 | 61 ± 18 |

All cultures were grown in LB medium and induced at time-point 0 (T = 0) with MMC. Values are the average of three replicas (Miller units ± standard deviation).

**Table S5B: β-galactosidase data in Miller units for Figure 3B.**

| Genotype | Strain | - MMC | + MMC |
| --- | --- | --- | --- |
| *P_sigN_^UP^-lacZ*  ΔpBS32 | DK5657 | 2 ± 1 | 105 ± 13 |
| *P_sigN_^DN^-lacZ*  ΔpBS32 | DK5658 | 9 ± 2 | 4 ± 1 |

All cultures were grown in LB medium and induced with mitomycin C for 1 hr. All values are the average of three replicas (Miller units ± standard deviation).

**Table S5C: β-galactosidase data in Miller units for Figure 3C**

| Reporter | WT | *lexA* |
| --- | --- | --- |
| *P_sigN_^UP^-lacZ* | 4 ± 1 (DK7291) | 171 ± 54  (DK7259) |
| *P_sigN_^DN^-lacZ* | 3 ± 1  (DK7292) | 8 ± 2  (DK7260) |

All cultures were grown in LB medium and cells were harvested once they reached mid-log phase. All values are the average of three replicas (Miller units ± standard deviation). Strains used to generate the data are indicated in parentheses.

**Table S5D: β-galactosidase data in Miller units for Figure 3D**

| Reporter | Strain | - IPTG | + IPTG |
| --- | --- | --- | --- |
| *P_sigN_^UP^-lacZ* | DK5657 | 6 ± 2 | 4 ± 1 |
| *P_sigN_^DN^-lacZ* | DK5658 | 18 ± 6 | 1013 ± 160 |

All cultures were grown in LB medium and induced by the addition of 1 mM IPTG for 1 hr. All values are

the average of three replicas (Miller units ± standard deviation).

**Table S5E: β-galactosidase data in Miller units for Figure 3E**

| Reporter | Strain | - IPTG | + IPTG |
| --- | --- | --- | --- |
| *P_zpcJ_-lacZ* | DK5968 | 15 ± 1 | 977 ± 24 |
| *P_zpcX_-lacZ* | DK5969 | 17 ± 8 | 925 ± 87 |
| *P_zpdG_-lacZ* | DK5970 | 8 ± 2 | 2353 ± 584 |

All cultures were grown in LB medium and induced with the addition of IPTG for 1 hr. All values are the average of three replicas (Miller units ± standard deviation).

**Table S5F: β-galactosidase data in Miller units for Figure S1A**

| Reporter | Strain | - IPTG | + IPTG |
| --- | --- | --- | --- |
| *P_repN_-lacZ* | DK4949 | 18 ± 3 | 16 ± 3 |
| *P_alfA_-lacZ* | DK4948 | 86 ± 26 | 3 ± 0.16 |
| *P_comI_-lacZ* | DK4725 | 34 ± 7 | 31 ± 15 |
| *P_sigN_-lacZ* | DK4943 | 17 ± 1 | 645 ± 125 |
| *P_zpbK_-lacZ* | DK4994 | 57 ± 2 | 50 ± 5 |
| *P_repN_-lacZ*  ΔpBS32 | DK4670 | 19 ± 5 | 26 ± 3 |
| *P_alfA_-lacZ*  ΔpBS32 | DK4669 | 357 ± 35 | 364 ±51 |
| *P_comI_-lacZ*  ΔpBS32 | DK4671 | 44 ± 6 | 49 ± 7 |
| *P_sigN_-lacZ*  ΔpBS32 | DK4401 | 4 ± 0 | 36 ± 2 |
| *P_zpbK_-lacZ*  ΔpBS32 | DK4673 | 1 ± 1 | 1 ± 1 |

All cultures were grown in LB medium and induced with the addition of IPTG for 1 hr. All values are the average of three replicas (Miller units ± standard deviation).
