# Supplementary material for: Transcriptional Regulation and Mechanism of SigN (ZpdN), a pBS32-Encoded Sigma Factor in Bacillus subtilis
Source: mBio. 2019 Sep 17;10(5):e01899-19. doi: 10.1128/mBio.01899-19 (PMC6751061; doi:10.1128/mBio.01899-19)
Supplement: TABLE S4 [file mBio.01899-19-st004.docx]

**Table S4: Rend-seq data of chromosomal genes**

| Gene | Fold increase^a^ | Annotation |
| --- | --- | --- |
| *clpE* | 13 | ClpP unfoldase |
| *czcD* | 36 | K^+^/H^+^ exchange transporter |
| *czcO* | 23 | monooxygenase |
| *glgP* | 15 | glycogen phosphorylase |
| *manA* | 27 | mannose 6-P isomerase |
| *manP* | 27 | mannose PTS EII |
| *rocB* | *** | arginine utilization |
| *sspF* | 79 | DNA compaction during sporulation |
| *ybcL* | 13 | unknown |
| *yrpB* | 11 | unknown |
| *yrzI* | 31 | unknown |
| *yvdR* | 47 | similar to multidrug efflux pump |
| *yvdS* | 118 | similar to multidrug efflux pump |
| *yvdT* | 15 | similar to transcriptional regulator |
| *yxcD* | 103 | unknown |

Fold change is the read count density of the SigN overexpression state (DK1634 induced for 1 hr with 1 mM IPTG) divided by the read count density of the wild type (DK607) for each gene. *** indicates that reads were detected in the *sigN* induced state but not in the wild type and division by zero is impossible.
