# Supplementary material for: Transcriptional Regulation and Mechanism of SigN (ZpdN), a pBS32-Encoded Sigma Factor in Bacillus subtilis
Source: mBio. 2019 Sep 17;10(5):e01899-19. doi: 10.1128/mBio.01899-19 (PMC6751061; doi:10.1128/mBio.01899-19)
Supplement: TABLE S1 [file mBio.01899-19-st001.docx]

**Table S1: Plasmids**

| Plasmid | Genotype |
| --- | --- |
| pATB9 | *thrC::P_sigN_^UP^-lacZ mls amp* |
| pATB10 | *thrC::P_sigN_^DN^-lacZ mls amp* |
| pATB11 | *his-SUMO-lexA amp cat* |
| pATB12 | *thrC::P_zpcJ_-lacZ mls amp* |
| pATB13 | *thrC::P_zpcX_-lacZ mls amp* |
| pATB14 | *thrC::P_zpdG_-lacZ mls amp* |
| pBM05 | *his-SUMO-sigN amp cat* |
| pDG1663 | *thrC::lacZ mls amp* |
| pDP476 | *thrC::P_alfA_-lacZ mls* |
| pDP477 | *thrC::P_repN_-lacZ mls* |
| pDP478 | *thrC::P_comI_-lacZ mls* |
| pDP480 | *thrC::P_zpbK_-lacZ mls* |
| pTB146 | *6His-SUMO amp* |
