# Supplementary material for: Transcriptional Regulation and Mechanism of SigN (ZpdN), a pBS32-Encoded Sigma Factor in Bacillus subtilis
Source: mBio. 2019 Sep 17;10(5):e01899-19. doi: 10.1128/mBio.01899-19 (PMC6751061; doi:10.1128/mBio.01899-19)
Supplement: TABLE S2 [file mBio.01899-19-st002.docx]

**Table S2: Primers**

| Primer | Sequence |
| --- | --- |
| 4438 | cacagcttggttctaagaatttg |
| 4441 | gcttgttcagcatcgatacc |
| 4498 | tcatgtttgacagcttatcatcg |
| 4500 | cgatgataagctgtcaaacatggtagttcaagagttcaatctttc |
| 4501 | gaaagattgaactcttgaactaccatgtttgacagcttatcatcg |
| 4527 | cccagcttgaattgatacacta |
| 4528 | tagtgtatcaattcaagctgggtctatattgctatttcatgttgg |
| 4617 | ctcctggatccttaagcgatctcaaggtattctttgg |
| 4665 | aggaggctcttctggtatgaagaagaaagaaatcttattttc |
| 4707 | tagtgtatcaattcaagctgggaggattatgaaagtttagtttgttttg |
| 5048 | aggaggaattcgaataccttgagatcgcttaacataa |
| 5049 | aggagggatccgttaaagcatctccttagttttcttta |
| 5050 | aggagggaattccggaagaatatgtagaaga |
| 5051 | aggaggggatccttttgttgctcctttttgttgaagtc |
| 5052 | aggaggaattctactgtagtatttttgtagagaagtcg |
| 5053 | aggagggatccattctcacctccttctttctagaca |
| 5122 | aggaggaattccttaaccactgaactagagata |
| 5123 | ctcctggatccggcgtactcggtaggattc |
| 5661 | cgcatttggatacatcgattg |
| 5662 | caattcgccctatagtgagtcgtttttgcagaatgtcagcattcta |
| 5663 | ccagcttttgttccctttagtgagaacctctgctttaataaaacgg |
| 5664 | cagcaaacgtcagtttattcg |
| 6087 | aggaggaattcgtgtacgatctagtacttatgt |
| 6088 | aggagggatcctctatattgctatttcatgttgg |
| 6089 | aggaggaattctacctccttttcttttgctaaat |
| 6090 | aggagggatccataagtactagatcgtacacct |
| 6252 | cagcacattatcctcctaaga |
| 6253 | cctgacgatcactcattctat |
| 6276 | aggaggaattcatagatgaaaaatccatataaagtt |
| 6277 | aggagggatcccctccgatttgtttaatgtgat |
| 6278 | aggagggatcctattcctccgatttaaatatggt |
| 6279 | aggaggaattcgctacatcatcgacaccagc |
| 6283 | aaatattttacagtgttccctcaatcaccgtat |
| 6284 | ggaacactgtaaaatattttttagcgaataagaacgg |
